# Tidal day organic and inorganic material flux of ponds in the Liberty Island freshwater tidal wetland

**DOI:** 10.1186/s40064-015-1068-6

**Published:** 2015-06-17

**Authors:** Peggy W Lehman, Shawn Mayr, Leji Liu, Alison Tang

**Affiliations:** Division of Environmental Services, California Department of Water Resources, 3500 Industrial Blvd, West Sacramento, CA 95691 USA; North Central Regional Office, California Department of Water Resources, 3500 Industrial Blvd, West Sacramento, CA 95691 USA

**Keywords:** Freshwater tidal wetland, Wetland material flux, Vegetated ponds, Carbon flux

## Abstract

The loss of inorganic and organic material export and habitat produced by freshwater tidal wetlands is hypothesized to be an important contributing factor to the long-term decline in fishery production in San Francisco Estuary. However, due to the absence of freshwater tidal wetlands in the estuary, there is little information on the export of inorganic and organic carbon, nutrient or phytoplankton community biomass and the associated mechanisms. A single-day study was conducted to assess the potential contribution of two small vegetated ponds and one large open-water pond to the inorganic and organic material flux within the freshwater tidal wetland Liberty Island in San Francisco Estuary. The study consisted of an intensive tidal day (25.5 h) sampling program that measured the flux of inorganic and organic material at three ponds using continuous monitoring of flow, chlorophyll *a*, turbidity and salt combined with discrete measurements of phytoplankton community carbon, total and dissolved organic carbon and nutrient concentration at 1.5 h intervals. Vegetated ponds had greater material concentrations than the open water pond and, despite their small area, contributed up to 81% of the organic and 61% of the inorganic material flux of the wetland. Exchange between ponds was important to wetland flux. The small vegetated pond in the interior of the wetland contributed as much as 72–87% of the total organic carbon and chlorophyll *a* and 10% of the diatom flux of the wetland. Export of inorganic and organic material from the small vegetated ponds was facilitated by small-scale topography and tidal asymmetry that produced a 40% greater material export on ebb tide. The small vegetated ponds contrasted with the large open water pond, which imported 29–96% of the inorganic and 4–81% of the organic material into the wetland from the adjacent river. This study identified small vegetated ponds as an important source of inorganic and organic material to the wetland and the importance of small scale physical processes within ponds to material flux of the wetland.

## Background

Freshwater wetlands are generally thought to be a sink for nutrients and a source of particulate and dissolved organic carbon to adjacent rivers and lakes (Junk et al. 1989; Bouchard 2007). Total nitrogen and phosphorus were reduced by 79 and 88% after passing through the Amelia wetland in Louisiana (Day et al. 2006). In the Patuxent River Estuary, freshwater tidal wetlands removed 46–76% of the nitrogen and phosphorus load (Boynton et al. 2008). The Vaccarés lagoon wetlands in the Rhone River watershed removed over 95% of the nitrogen and phosphorus imported and exported 5–6 times more organic carbon than the wetlands imported (Chauvelon 1998). Organic carbon production in wetlands is facilitated by elevated net primary productivity of phytoplankton in shallow waters, where the euphotic zone to mixed depth ratio is high (Heip et al. 1995) and phytoplankton cells accumulate due to long residence time (Hein et al. 1999). Inorganic and organic material flux is highly variable among wetlands. Both the magnitude and direction of material flux can vary by many fold, even in adjacent wetlands (Arrigoni et al. 2008).

The quantity and quality of material exported from wetlands is influenced by internal and external physical, chemical and biological processes. External factors such as climate affect the magnitude of water flow and physical factors like water temperature and depth (Childers et al. 2000). Internal factors such as primary productivity, benthic recycling and denitrification affect the quantity and quality of nutrients and organic material available for export (Childers et al. 2000; Childers 2006). Sedimentation can also remove nutrients or organic matter from the water column (Moustafa 1999). The amount of inorganic and organic material exported from the wetland is further affected by wetland morphology, velocity heterogeneity and vegetation type that control the speed and direction of material movement in the wetland (McKellar et al. 2007; Lightbody et al. 2008).

Tide is also a major factor affecting material transport in freshwater tidal wetlands. For the Liberty Island freshwater tidal wetland in San Francisco Estuary (SFE), California, over 90% of the inorganic and organic material flux of open water habitat was tidal (Lehman et al. 2010). Inorganic and organic material flux in the summer was similarly influenced by tidal asymmetry in Mildred Island a flooded island in the delta region of SFE (Ganju et al. 2005; Lopez et al. 2006). Tidal impacts can be subtle in SFE wetlands. Carbon production increased on a fortnightly basis with the number of daylight hours that overlapped ebb tide (Lucas et al. 2006).

It is hypothesized that the long-term loss of over 90% of the wetland habitat in SFE reduced food resources and habitat needed to support estuarine fish production (Brown 2003). Estuarine production is a concern in SFE where there has been a decline in biomass and shift in species composition at all levels of the aquatic food web over time, including a downward shift in the production of phytoplankton, zooplankton and fish, particularly since 2000 (Lehman 2000, 2004; Kimmerer 2004; Sommer et al. 2007). The importance of freshwater tidal wetlands to fishery production in SFE is suggested by the abundance of native fish species in areas with adjoining freshwater tidal wetland habitat in northern SFE near the freshwater tidal wetland, Liberty Island (Sommer et al. 2001; Nobriga et al. 2008). Similarly, fishery production in the Everglades and Chesapeake Bay was also affected by the quantity and quality of nutrients and organic matter produced in adjoining wetlands (Childers et al. 2000; Childers 2006).

For SFE, modeling studies suggested that wetland and floodplain habitats with a high euphotic zone depth to mixed depth ratio could be a source of organic carbon (Jassby and Cloern 2000). Modeling studies also suggested high residence time and warmer water temperature enhanced chlorophyll *a* production in Yolo Bypass, a large floodplain in SFE (Sommer et al. 2004). Field studies further confirmed that 14–37% of the total chlorophyll *a* or diatom plus green algal carbon exported to SFE from the Sacramento River and Yolo Bypass floodplain was produced in the floodplain (Lehman et al. 2008). Wetlands in flooded islands within the delta region of SFE are also a source of chlorophyll *a* and suspended sediment during the spring and summer (Lopez et al. 2006). Much less is known about freshwater tidal wetlands in SFE. Recent research demonstrated that both inorganic and organic materials as well as phytoplankton and zooplankton carbon were periodically exported to river channels in SFE from the Liberty Island freshwater tidal wetland (Lehman et al. 2010). However, the factors within the wetland that influence the magnitude and direction of material flux are unknown. The lack of information is partially due to the difficulty of sampling in shallow freshwater tidal wetlands, where conditions change rapidly with the tide and environmental conditions and shallow water, thick mud, dewatering, remote access and human and animal disturbance impede sampling.

**Figure 1:**
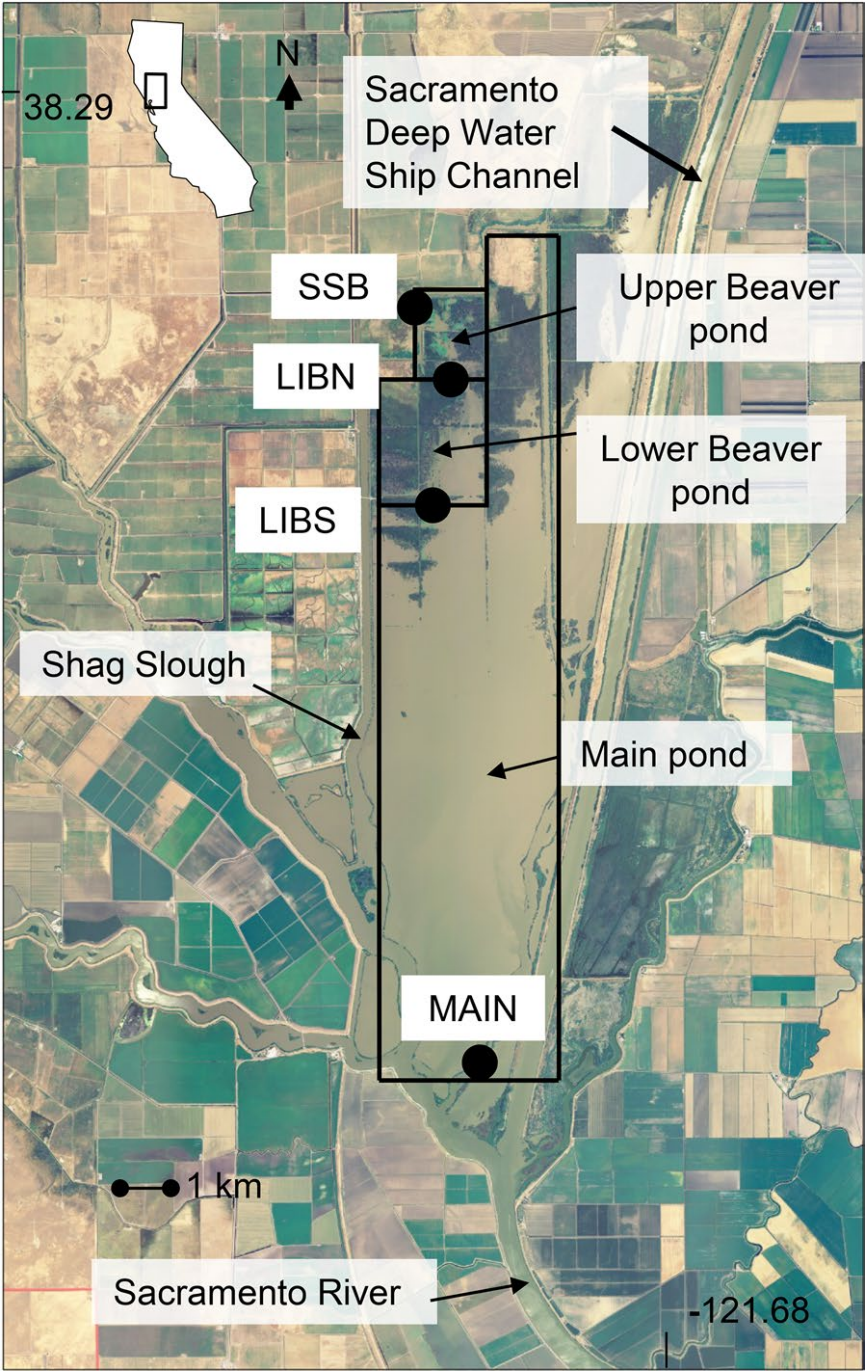
Map of Liberty Island indicating the location of ponds and sampling stations.

The purpose of this initial study was to test the hypothesis that small shallow vegetated ponds are a source of inorganic and organic material, including phytoplankton biomass, to the freshwater tidal wetland. This hypothesis was addressed by conducting an intensive 25.5 h tidal day study that measured the phytoplankton community biomass, inorganic and organic carbon, suspended sediment, chloride and nutrient flux and associated physical and chemical factors among two small vegetated ponds (Upper and Lower Beaver Pond) and one large open water pond (Main Pond) in Liberty Island, SFE (Figure 1). Due to rapid changes associated with tide, material flux was determined with a combination of continuous monitors and high frequency discrete sampling at 1.5 h intervals. Information from this study will provide valuable insight into the potential contribution of small ponds to freshwater tidal wetland material production and export needed to design future long-term wetland research in SFE.

## Results

### Hydrodynamics

**Figure 2:**
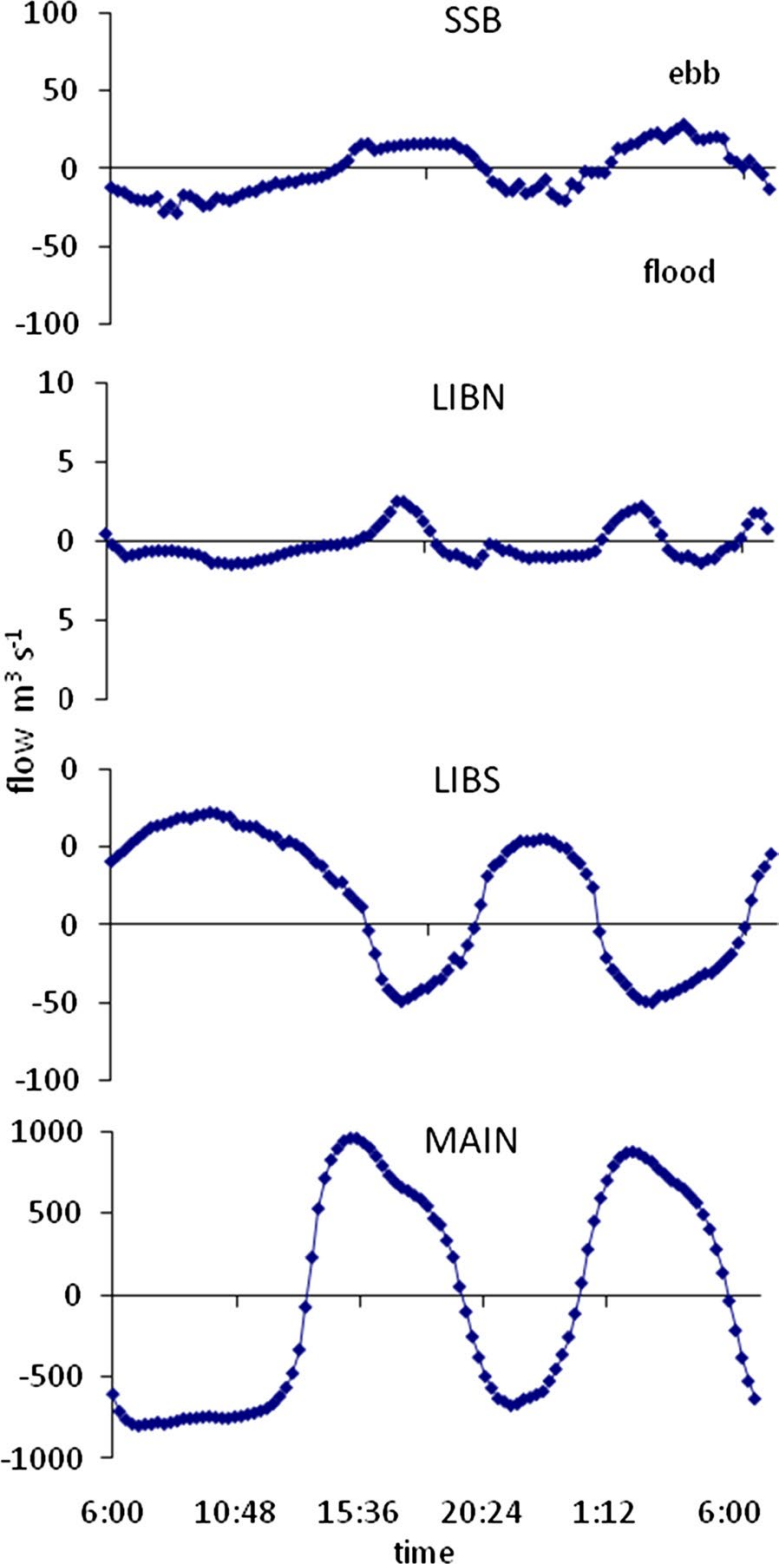
Flow measured at stations in Liberty Island on 12 July and 13 July 2006. *Positive values* indicate southward while *negative values* indicate northward flow. n = 102 for each station.

The magnitude and timing of water flow within the wetland was strongly influenced by water exchange with the river channels. At stations SSB and MAIN, which were connected to the river channel, water flow varied directly with the semi-diurnal tide (Figure 2). In contrast, water flow at stations LIBN and LIBS, in the interior of the wetland, did not vary directly with the tide. The flow at LIBN was out of phase with that at SSB by about an hour. At LIBS the peak flow differed by up to 6 h from MAIN. The connectivity with the river also affected the magnitude of the water flow at each station (Figure 2). Average water flow was over an order of magnitude greater at MAIN, which was connected to the large Sacramento River, than at SSB, which was connected to the small Shag Slough. The average water flow also decreased with distance from the river channel. Water flow at LIBN was an order of magnitude lower than at SSB, 30 times lower than at LIBS and over two orders of magnitude lower than at MAIN. Water flow at LIBS was greater than at LIBN because of its closer proximity to the Sacramento River, but was still an order of magnitude lower than at MAIN, which was directly connected to the Sacramento River.

**Figure 3:**
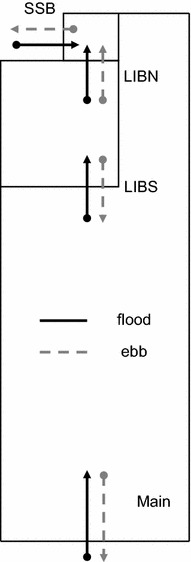
Schematic of tidal flow in Liberty Island during ebb and flood tide.

The direction of water flow in the wetland was also influenced by tidal asymmetry and small-scale topography. On flood tide, water initially flowed east into the wetland from Shag Slough at SSB and south past LIBN. Water also flowed north from the Sacramento River past MAIN and LIBS. However, as the flood tide progressed, the large flow northward past MAIN shifted the flow at LIBN northward into Upper Beaver Pond and resulted in a net flow of water northward (Figure 3). On ebb tide, the water flow was bi-directional from the interior of the wetland. Water initially flowed west at SSB and south at both LIBN and LIBS due to the strong influence of ebb tide from Shag Slough and the Sacramento River. However, as ebb tide progressed the direction of the water flow in Lower Beaver Pond split with some water flowing north past LIBN to SSB and the rest flowing south past LIBS (Figure 3). This split in the flow pattern was caused by a geographical high spot in the northern quadrant of Lower Beaver Pond which is dry at low water on ebb tide. As a result, the upper and lower sections of Lower Beaver Pond were hydraulically isolated at low water and the strong influence of the Sacramento River flow on the upper portion of the wetland was removed. This flow pattern created a shift in the tidal phase in the interior of the wetland such that water flow at LIBN was characterized by a shorter period of high flow and greater hourly variability than at SSB or MAIN (Figure 2).

### Water quality conditions

Water quality conditions varied significantly (p < 0.01) between vegetated and open water ponds (Table 1). Water temperature was greater at SSB, LIBN and LIBS than MAIN. Specific conductance was greater in the northern portion of the wetland at SSB and LIBN than in the southern portion of the wetland at LIBS and MAIN; the reverse occurred for pH. Turbidity was lower at MAIN while dissolved oxygen was lower at SSB than all other stations.

**Table 1:** Mean and standard deviation water quality conditions measured at 1.5 h intervals over 25.5 h for each pond in Liberty Island wetland between 12 July and 13 July 2006

Station	Water temperature (°C)	Specific conductance (µS cm^−1^)	Dissolved oxygen (mg l^−1^)	pH	Turbidity (NTU)
SSB	23 ± 1 a	389 ± 12 c	7.0 ± 0.7 a	7.7 ± 0.1 a	84 ± 18 a
LIBN	23 ± 4 a	356 ± 65 c	9.4 ± 1.6 b	7.8 ± 0.4 a	126 ± 44 a
LIBS	23 ± 4 a	254 ± 41 b	9.0 ± 1.3 b	8.2 ± 0.2 b	95 ± 38 a
MAIN	21 ± 1 b	187 ± 8 b	8.9 ± 0.6 b	8.1 ± 0.1 b	39 ± 5 b

Values with similar letters were not significantly different at the 0.05 level or higher. n = 18 for each station.

### Material concentration

**Figure 4:**
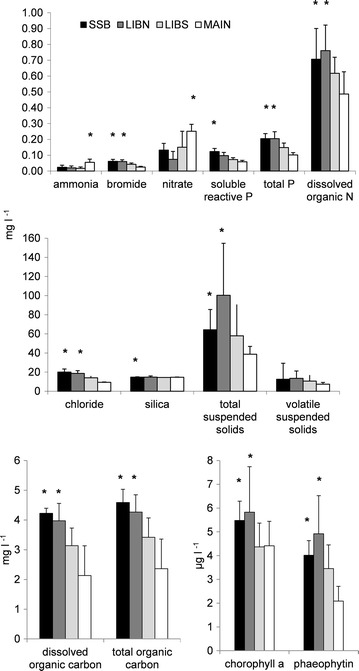
Tidal day average (*bar*) and standard deviation (*line*) of material concentrations measured at stations in Liberty Island on 12 July and 13 July 2006. Significantly different concentrations at the p < 0.05 level or higher are indicated by an *asterisk* (∗).

The concentration of most inorganic and organic material was greater in the vegetated ponds (Figure 4). Upper Beaver Pond at SSB and LIBN had greater (p < 0.05) concentrations of most inorganic and organic materials than Lower Beaver Pond at LIBS and Main Pond at MAIN, including total phosphorus, dissolved organic nitrogen, chloride, total suspended solids, total and dissolved organic carbon, chlorophyll *a* and phaeophytin. The greatest difference in material concentration occurred between LIBN and MAIN, where concentrations often differed by a factor of 2. Greater (p < 0.05) average nitrate and ammonium concentration at MAIN than the other stations was also accompanied by a relatively greater nitrogen to phosphorus molar ratio of 12, compared with SSB (3), LIBN (2) and LIBS (5), respectively.

**Figure 5:**
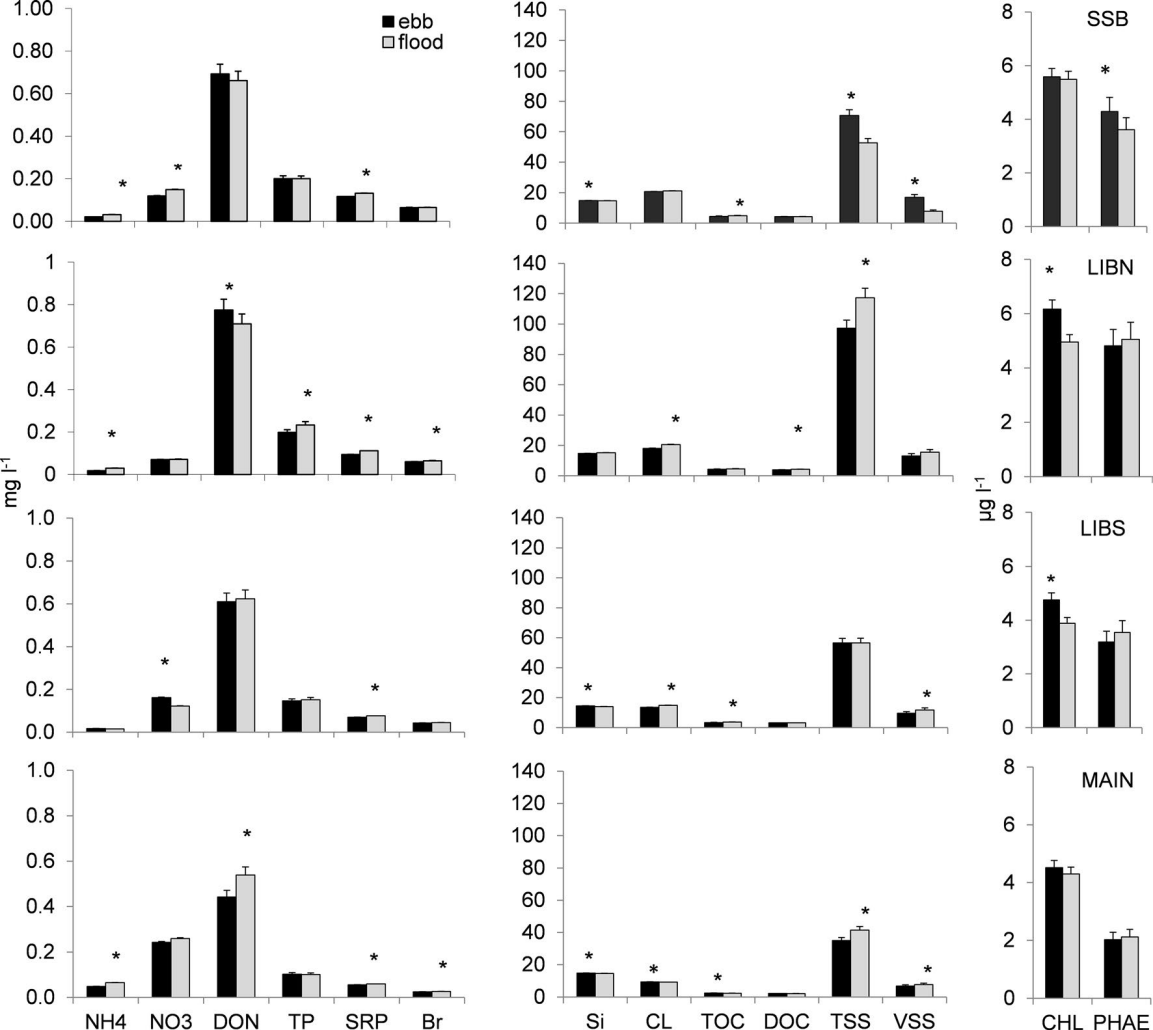
Average (*bar*) and standard deviation (*line*) of inorganic and organic material concentrations measured on ebb and flood tide at stations in Liberty Island on 12 July and 13 July 2006. Significantly different concentrations at the p < 0.05 level or higher are indicated by an *asterisk* (∗). n = 102 for each station.

Material concentration among stations varied with tide. On ebb tide, chlorophyll *a* concentration was greater (p < 0.05) at the interior stations LIBS and LIBN than other stations, while TOC was greater (p < 0.05) for the channel station at MAIN (Figure 5). Among the nutrients, silica concentration was greater (p < 0.05) for LIBS, MAIN and SSB, while chloride concentration was greater (p < 0.05) at MAIN than other stations on ebb tide. Total and volatile suspended solids were only greater (p < 0.01) among stations on ebb tide at SSB. On flood tide, both soluble reactive phosphorus (p < 0.01) and ammonium (p < 0.05) concentrations were greater at all stations except LIBS. More total suspended solids (p < 0.01) occurred on flood tide at MAIN, while more volatile suspended solids (p < 0.05) occurred at both LIBS and MAIN. Chloride concentration was also greater (p < 0.01) on flood tide in the interior of the wetland at LIBS and LIBN.

Phytoplankton carbon differed among ponds and with tide (not shown). Average green (525 ± 1,222 pg C ml^−1^) and miscellaneous flagellate (3,686 ± 9,198 pg C ml^−1^) carbon were greater (p < 0.01) at MAIN than other stations. However, there was no statistical difference among stations for diatom or cyanobacterial carbon (1,893 ± 2,345 and 328 ± 576 pg C ml^−1^). More diatom carbon (p < 0.05) on ebb tide at SSB (1,393 ± 3,768 pg C ml^−1^) and on flood tide at LIBN (16,276 ± 17,321 pg C ml^−1^), suggested diatoms were produced in the vegetated ponds. Maximum cyanobacteria carbon was detected in the interior of wetland on flood tide in Lower Beaver Pond at LIBN (12,050 ± 742 pg C ml^−1^) and LIBS (4,366 ± 8,515 pg C ml^−1^). In contrast, maximum (p < 0.05) miscellaneous flagellate carbon occurred on ebb tide at LIBN (1,577 ± 3,706 pg C ml^−1^).

### Material flux

**Figure 6:**
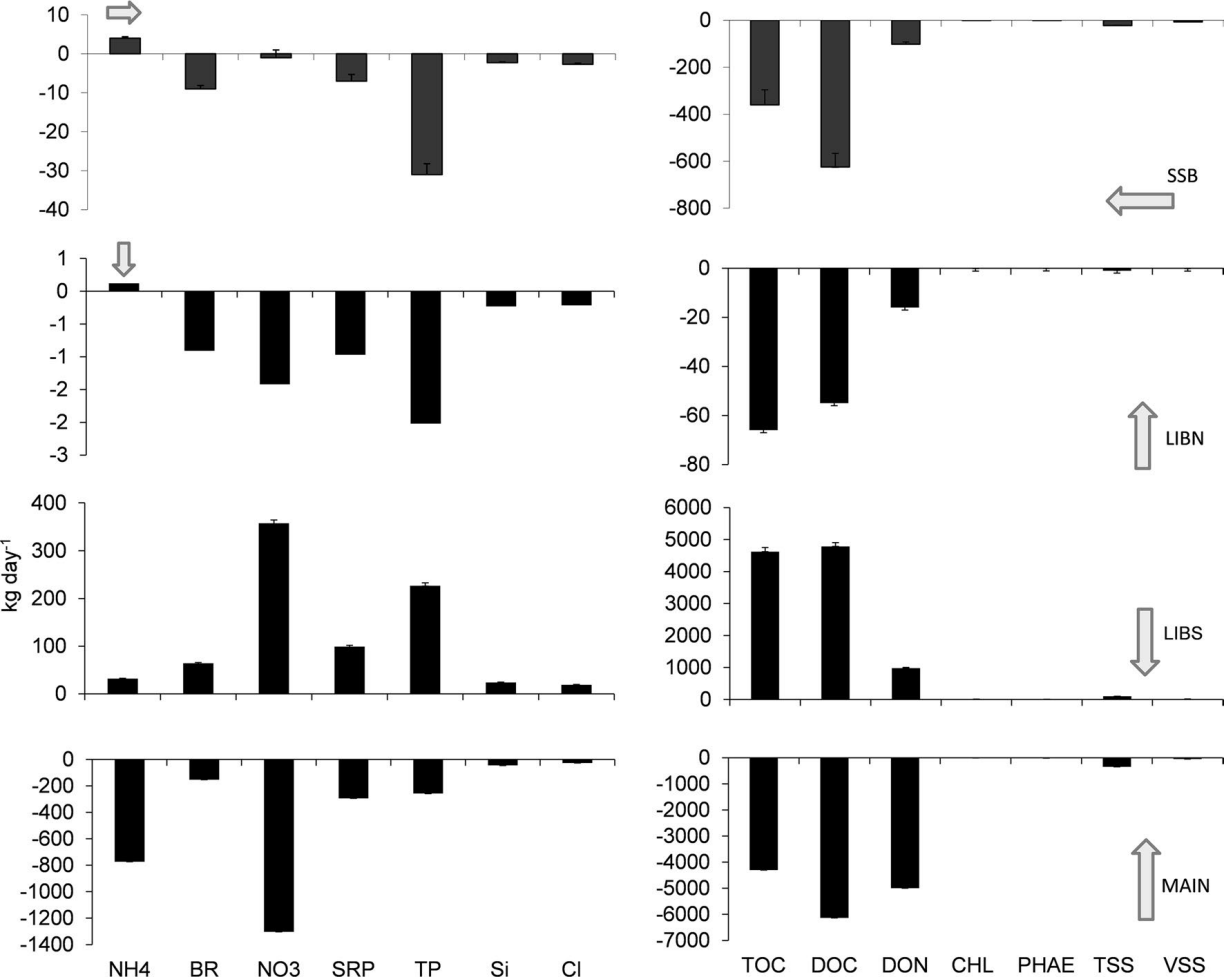
Tidal day inorganic and organic material flux at each station measured for Liberty Island on 12 July and 13 July 2006. *Positive flux values* indicate net movement of material from north to south at LIBN, LIBS or MAIN and west to east at SSB; *negative flux values* indicate the reverse. n = 102 for each station.

Vegetated ponds produced and exported both inorganic and organic material to adjacent ponds and the river. The negative inorganic and organic material flux at SSB indicated that most of the materials in Upper Beaver Pond were exported westward into Shag Slough (Figure 6). Lower (p < 0.05) negative (northward) material flux past LIBN than SSB further suggested that the exported inorganic and organic material at SSB was produced in Upper Beaver Pond and not imported from Lower Beaver Pond. Lower Beaver Pond also exported inorganic and organic material into Main Pond at LIBS. The large positive (southward) material flux at LIBS, but small negative material flux at LIBN indicated Lower Beaver Pond was a source of inorganic and organic material to Main Pond. The relatively small positive flux at LIBS and large negative flux at MAIN further indicated most inorganic and organic material was imported into Main Pond from the Sacramento River. In fact, the inorganic and organic material flux at MAIN was greater (p < 0.05) than the material flux from SSB, LIBN or LIBS by 1 to many orders of magnitude for most inorganic and organic materials, except total organic carbon and chlorophyll *a*.

The vegetated ponds were the primary source of total organic carbon and chlorophyll *a* within the wetland and to the adjacent rivers. The total organic carbon and chlorophyll *a* flux at SSB, LIBN and LIBS combined was greater than the flux of total organic carbon and chlorophyll *a* at MAIN (Figure 6). Most of the total organic carbon (4,624 kg day^−1^) and chlorophyll *a* (10 kg day^−1^) flux occurred at LIBS, which was also the major source of total organic carbon and chlorophyll *a* to Main Pond. The significantly (p < 0.05) lower and negative chlorophyll *a* flux at LIBN compared with the large positive chlorophyll *a* flux at LIBS indicated that the total organic carbon and chlorophyll *a* exported from Lower Beaver Pond at LIBS was produced within the pond and not imported from Upper Beaver Pond at LIBN. The relative health of the phytoplankton could not account for the greater chlorophyll *a* flux into Main Pond from Lower Beaver Pond. Both average chlorophyll *a* concentration and the percent chlorophyll *a* to total pigment (chlorophyll *a* plus phaeophytin) ratio was similar for Main Pond and Lower Beaver Pond (chlorophyll *a* 4.4 ± 1.0 and 4.4 ± 1.5 µg l^−1^ and percent chlorophyll *a* to total pigment 68 ± 9 and 58 ± 12%, respectively; Figure 4).

The river was the primary source of ammonium to the wetland. Ammonium entered the wetland at SSB from Shag Slough and at MAIN from the Sacramento River (Figure 6). The positive flux at SSB and negative flux at LIBN, combined with the relatively small difference between the nitrate flux at SSB and LIBN, suggested 97% of the ammonium imported at SSB was retained in Upper Beaver Pond, and not exported as either ammonium or nitrate. The greater negative ammonium flux at MAIN than the positive ammonium flux at LIBS, indicated that most of the ammonium in Main Pond was imported from the Sacramento River. The greater import of nitrate than ammonium at MAIN also suggested most of the nitrate in Main Pond was imported from the Sacramento River and not produced by the oxidation of ammonium within the pond.

**Figure 7:**
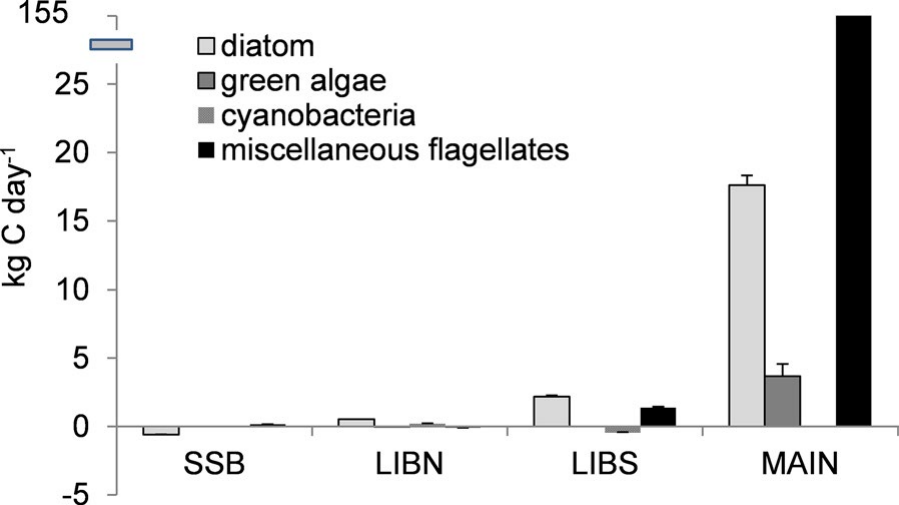
Tidal day phytoplankton and cyanobacteria carbon flux (*bar*) and standard deviation (*line*) by taxa for stations measured in Liberty Island wetland on 12 July and 13 July 2006. n = 102 for each station.

Phytoplankton and cyanobacteria flux affected the carbon distribution and export of primary producers into ponds and the adjacent rivers. Most of the carbon flux was associated with diatoms and miscellaneous flagellates, which accounted for 2 and 97% of the carbon flux, respectively. Close to 3% of the total diatom carbon flux occurred at SSB. Like most organic material, diatom carbon (−0.58 kg C day^−1^) was exported into Shag Slough, but it was accompanied by a small (0.16 kg C day^−1^) import of miscellaneous flagellate carbon (Figure 7). Positive diatom carbon flux at LIBN, LIBS and MAIN indicated that diatoms moved southward within the wetland into Lower Beaver Pond, then to Main Pond and finally into the Sacramento River. The resulting export of diatom carbon at MAIN accounted for 84% of the diatom carbon flux in the wetland. A positive flux at LIBN but negative flux at LIBS, suggested cyanobacteria carbon was retained in Lower Beaver Pond. Positive flux at LIBS and an even larger positive flux at MAIN of miscellaneous flagellate carbon, indicated the wetland was a source of miscellaneous flagellates to the Sacramento River. In fact, 97% of the miscellaneous flagellate carbon flux (155 kg day^−1^) in the wetland occurred at MAIN. The miscellaneous flagellate carbon flux at MAIN was accompanied by a small export of green algae carbon (3.7 kg day^−1^).

The ponds differed in the direction and magnitude of material flux. Main Pond had the largest inorganic material flux, which ranged from −103 kg day^−1^ for total phosphorus to −267,456 kg day^−1^ for total suspended solids (Table 2). Main Pond accounted for 96% of the ammonium, 76% of the nitrate, 70% of the soluble phosphorus and 69% of the total suspended solids flux in the wetland. The negative inorganic material flux values for Main Pond indicated inorganic material moved northward into the wetland from the Sacramento River. Lower Beaver Pond usually accounted for less than half of the inorganic material flux of Main Pond, but contributed 52% of the chloride (18,113 kg day^−1^) and 61% of the total phosphorus (216 kg day^−1^) of the wetland. Unlike Main Pond, the inorganic material flux of Lower Beaver Pond was positive, which indicated a net southward movement of inorganic material through the wetland and into Main Pond. Like Main Pond, total suspended solids (96,106 kg day^−1^) had the largest material flux followed by silica (22,915 kg day^−1^) and then chloride in Lower Beaver Pond. Upper Beaver Pond had the lowest inorganic material flux and accounted for less than 10% of the inorganic material flux in the wetland. The largest inorganic material flux in Upper Beaver Pond was associated with total suspended solids (23,235 kg day^−1^), followed by chloride (−2,927 kg day^−1^) and silica (−2,515 kg day^−1^). Negative flux values also characterized Upper Beaver Pond, where the net movement of inorganic material was northward and then westward into Shag Slough. Only ammonium, with a positive flux of 4 kg day^−1^ was imported into the wetland through Upper Beaver Pond.

**Table 2:** Daily sum and standard deviation (parenthesis) of the hourly material flux and the percent of the total daily flux contributed by each pond, plus the total wetland material flux for inorganic and organic materials in Liberty Island wetland measured on July 12–13, 2006

Material	Upper Beaver Pond (kg day^−1^)	%	Lower Beaver Pond (kg day^−1^)	%	Main Pond (kg day^−1^)	%	Wetland (kg day^−1^)
Ammonium	4 (0.4)	1	30 (1)	4	−766 (35)	96	−732 (34)
Bromide	−9.7 (0.9)	5	60 (1.6)	34	−109 (14)	61	−58 (13)
Chloride	−2,926 (297)	8	18,113 (521)	52	−13,646 (5,119)	39	1,540 (4,877)
Nitrate	−2.6 (2.0)	<1	344 (7)	23	−1,127 (147)	76	−786 (140)
Soluble phosphorus	−7.4 (1.7)	2	93 (3)	28	−234 (33)	70	−148 (32)
Total phosphate	−32.5 (2.9)	9	216 (6)	61	−103 (57)	29	80 (54)
Silica	−2,514 (210)	4	22,915 (548)	41	−31,074 (8,319)	55	−10,674 (7,887)
Total suspended solids	−23,234 (950)	6	96,105 (2,887)	25	−267,456 (21,640)	69	−194,585 (19,968)
Volatile suspended solids	−6,949 (205)	11	12,851 (522)	21	−40,841 (4,009)	67	−34,939 (3,720)
Total organic carbon	−426 (66)	7	4,389 (130)	72	−1,291 (1,301)	21	2,672 (1,232)
Dissolved organic carbon	−680 (60)	8	4,563 (117)	57	−2,776 (1,186)	35	1,106 (1,127)
Dissolved organic nitrogen	−118 (10)	2	921 (24)	17	−4,316 (286)	81	−3,513 (269)
Chlorophyll *a*	−0.9 (0.08)	9	9.1 (0.2)	87	0.40 (2.6)	4	9 (2)
Phaeophytin	−1.2 (0.06)	9	4.8 (0.2)	37	−7.2 (1.2)	55	−4 (1)

n = 102 for each pond.

The net movement of organic material differed somewhat from the net movement of inorganic material in the wetland. High values characterized the volatile suspended solids and dissolved organic nitrogen flux in Main Pond and accounted for 67 and 81%, respectively, of the wetland flux for these materials (Table 2). Main Pond also accounted for 21–35% of the total and dissolved organic carbon flux of the wetland. Similar to the inorganic materials, the flux of total and dissolved organic carbon, volatile suspended solids and dissolved organic nitrogen was northward into the wetland from the Sacramento River. In contrast, the small and positive chlorophyll *a* flux of Main Pond accounted for only 4% of the chlorophyll flux (0.4 kg day^−1^) in the wetland. Greater and more positive chlorophyll *a* flux for Lower Beaver Pond than Main Pond indicated Main Pond received its chlorophyll *a* from Lower Beaver Pond. The greater negative phaeophytin flux than chlorophyll *a* flux for Main Pond and the reverse for Lower Beaver Pond also supported the contribution of live chlorophyll *a* from Lower Beaver Pond to Main Pond. Over all, Lower Beaver Pond accounted for 87% (9.1 kg day^−1^) of the chlorophyll *a* flux among the wetland ponds. Lower Beaver Pond also produced the majority of total organic carbon (72%) in the wetland, which again was exported southward into Main Pond. By contrast, Upper Beaver Pond accounted for only 2–11% of the organic material flux, with the largest flux associated with volatile suspended solids (−6,949 kg day^−1^). Like Lower Beaver Pond, the organic material flux in Upper Beaver Pond was characterized by negative values and indicated Lower Beaver Pond was a source of organic material to the river.

### Tidal asymmetry

**Figure 8:**
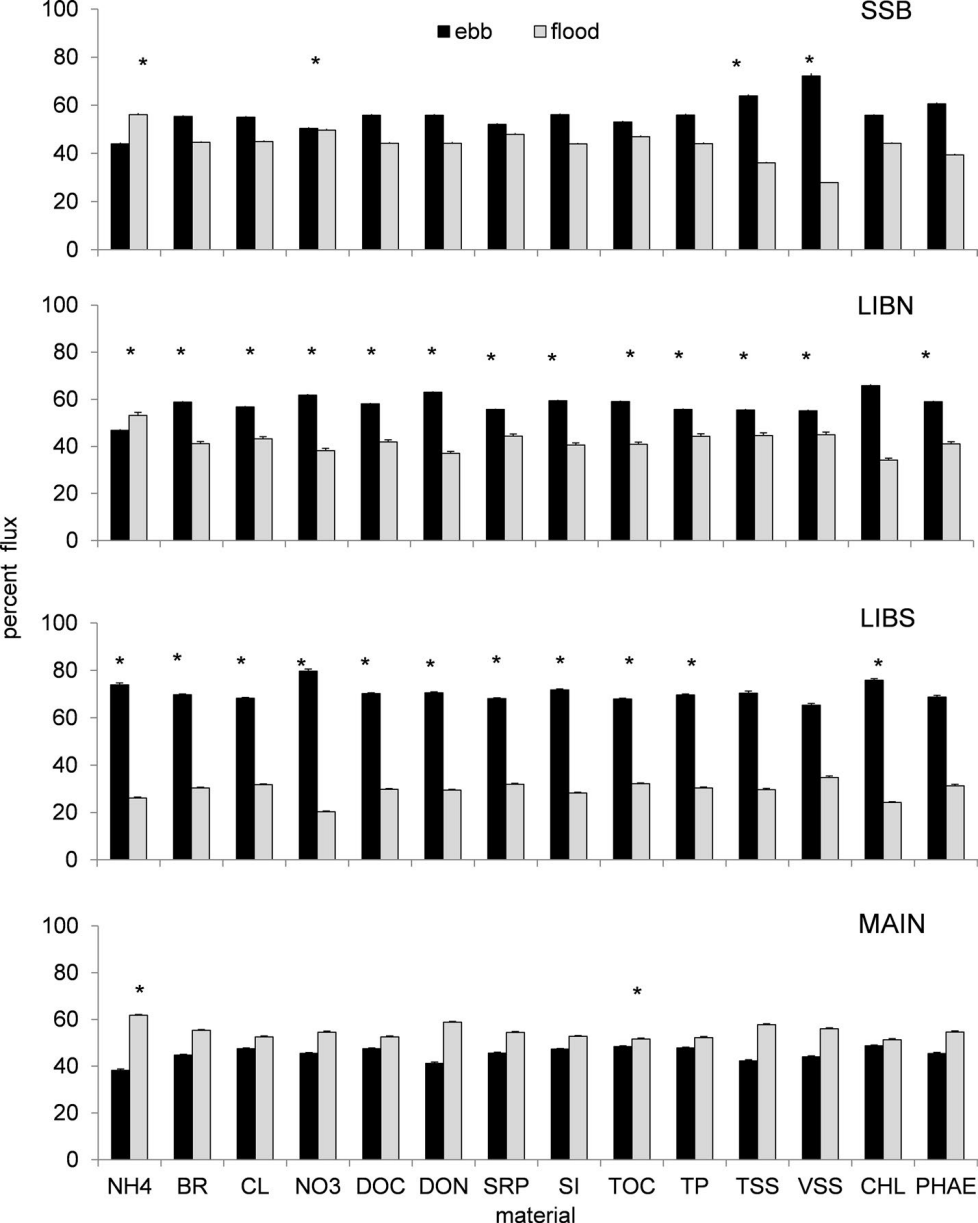
Average (*bar*) and standard deviation (*line*) of the percent inorganic and organic material flux measured on ebb and flood tide at stations in Liberty Island on 12 July and 13 July 2006. Significantly different concentrations at the p < 0.05 level or higher are indicated by an *asterisk* (∗). n = 102 for each station.

Tidal asymmetry in the interior of the wetland increased material export from the wetland. The percent material flux on ebb tide was greater (p < 0.05) for most materials in the interior of the wetland at LIBN and LIBS than at stations SSB and MAIN adjoining the river channel (Figure 8). The export of inorganic and organic material from the interior of the wetland was facilitated by both the greater material concentration (p < 0.05; Figure 5) and the 40% greater water flow (p < 0.01) at LIBS on ebb tide (Figure 2). About 71% of the water flow occurred at LIBS on ebb tide compared with only 60, 47 and 56% at LIBN, MAIN and SSB, respectively. The combined high flow and material concentration at LIBS on ebb tide produced a 40% greater (p < 0.05) flux on ebb than flood tide (70% ebb and 30% flood tide). Unexpectedly, there was no significant difference between tides in the percent material flux for most inorganic and organic materials at MAIN or SSB (range 42–55 and 45–58% for ebb and flood tide, respectively), despite the strong tidal influence of the river at these stations. Although the percent material flux varied more in the interior of the wetland, the absolute magnitude of the material flux was still greater at stations LIBS and MAIN closer to the large Sacramento River than stations LIBN and SSB near the small Shag Slough (not shown). However, similar to the percent material flux, greater (p < 0.05) material flux still occurred on ebb than flood tide. This was true even though more (p < 0.05) water was imported into the wetland past MAIN on flood tide.

## Discussion

**Figure 9:**
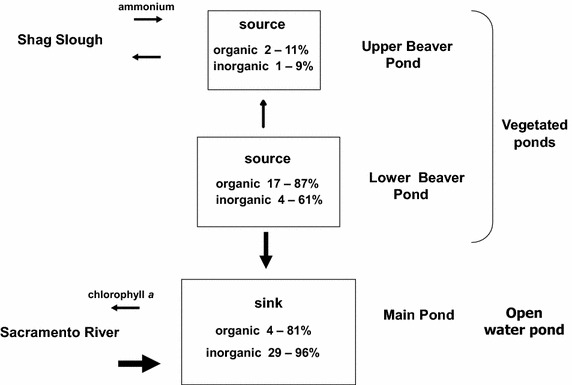
Diagram describing the percent contribution of each pond to the inorganic and organic material flux and the direction of the material flux within the wetland. *Arrows* indicate the direction of material movement and the relative magnitude of exchange.

Liberty Island wetland was a sink for most inorganic and organic materials over the tidal day. This was produced by the relatively large import of material into Main Pond from Lower Beaver Pond and the Sacramento River, compared with the relatively small export of material out of the wetland into Shag Slough from Upper Beaver Pond (Figure 9). Chlorophyll *a*, salt, nitrate and ammonium were similarly imported into Main Pond between 2004 and 2005 (Lehman et al. 2010). Wetlands are commonly a sink for inorganic nutrients that are taken up by aquatic plants or lost though sedimentation and mineralization (Saunders and Kalff 2001). Nitrogen and phosphorus loss in freshwater tidal marshes was also associated with denitrification, burial of particulate nitrogen and phosphorus and storage of nutrients in the water column and biota for the Patuxent River Estuary and Chesapeake Bay (Boynton et al. 2008). Material storage in the open water of Main Pond was probably facilitated by sedimentation which is increased when water depth is less than 1 m (Moustafa 1999). Hypoxic sediments can affect nitrogen removal through denitrification (McKellar et al. 2007), but this process was probably not important in Liberty Island where surface dissolved oxygen concentration was above saturation and the sediment layer is shallow due to compaction of soil from previous agricultural practices.

Lower Beaver Pond, a small vegetated pond in the interior of the wetland, was an important source of carbon and accounted for a large percentage of the total organic carbon and chlorophyll *a* flux within the wetland. Lower Beaver Pond contributed 87% of the chlorophyll *a*, 72% of the total organic carbon and 10% of the diatom flux among wetland ponds. The lack of water exchange between Lower Beaver Pond and the river may have been a critical factor contributing to the high export of carbon from Lower Beaver Pond to the other ponds, because it prevented export of carbon out of the wetland. Most of the organic matter in the freshwater tidal Shark River Slough in the Florida Coastal Everglades was produced in isolated bays (Xu et al. 2006). Algal biomass was also greater in pools with long residence time in 33 temporary freshwater pools in Hungary (Boven et al. 2008).

Both vegetated ponds combined exported 5–70% of the inorganic material exported by the wetland either to interior ponds or exterior river channels. Upper and Lower Beaver Ponds exported 31–60% of the total suspended solids, chloride and bromide and 5–70% of the major nutrients nitrate, ammonium, soluble phosphorus, total phosphorus and silica. Nitrogen, phosphorus, chloride and silica are often released from sediments in wetlands through plant uptake or leaching and decomposition of organic matter (Noe and Childers 2007; de Bettencourt et al. 2007). Main Pond also exported suspended sediment, soluble and total phosphorus in most seasons between 2004 and 2005 (Lehman et al. 2010). The amount of material exported by wetlands is partially dependent on the sedimentary source. Wetlands with terrigenous sediment sources like Liberty Island are characterized by greater material export than those with carbonate sediments like the Everglades (Sutula et al. 2003).

Increased tidal asymmetry coupled with wetland topography facilitated the inorganic and organic material export from Lower Beaver Pond in the interior of the wetland. During flood tide, high flows pushed water up and into the elevated region in the northern quadrat of Lower Beaver Pond. As the flood water receded, it left a pool of water that was isolated from the rest of Lower Beaver Pond by a sill, which prevented the pooled flood water from fully draining during ebb tide. The pooled flood water facilitated accumulation of inorganic material and the growth and accumulation of organic material. The pooled flood water and its accumulated inorganic and organic material was released back into the wetland during the next flood tide, when water was again pushed into the northern end of the pond and partially drained back into the wetland during ebb. The release of this pooled flood water back into the wetland produced a 40% increase in the organic and inorganic material export from Lower Beaver Pond on ebb tide. Most of the material flux also occurred on ebb tide for freshwater tidal bayous in the Atchafalaya River in Louisiana and freshwater tidal wetlands off the Hudson River (Stern et al. 1991; Arrigoni et al. 2008).

Vegetated ponds in freshwater tidal wetlands may function like floodplains along rivers that produce and store phytoplankton carbon until it is returned to the river with flood water (Junk et al. 1989; Tockner et al. 1999). Such processes commonly occur in floodplain habitat near Liberty Island. Long residence time during the drain phase of the flood-pulse cycle facilitated accumulation of more chlorophyll *a* concentration in the Yolo Bypass floodplain than the main Sacramento River channel (Lehman et al. 2008; Sommer et al. 2004). Carbon production in Yolo Bypass floodplain was also facilitated by an elevated growth efficiency and production to respiration ratio (Lehman et al. 2008). Elevated growth rate in the vegetated ponds was suggested by the net production of chlorophyll *a* concentration. Elevated nitrogen to phosphorus ratios may further enhance carbon production in vegetated ponds (Huang and Morris 2003). However, this was not the case for phytoplankton in Upper and Lower Beaver Ponds, where the nitrogen to phosphorus molar ratios were three times lower than the optimal Redfield N:P ratio of 16 and accompanied by relatively low chlorophyll *a* concentration. Higher vegetation may facilitate phytoplankton production in wetland ponds because of their influence on local physical conditions. Significantly greater water temperature, salinity and suspended sediment occurred in vegetated than open water ponds in Liberty Island. Local physical conditions were also affected by emergent vegetation in California and Georgia wetlands (Lightbody et al. 2008; Miller and Fujii 2010).

The daily tidal pulse of material created by the export of carbon from the vegetative ponds during the ebb tide and addition of nutrients needed to support future carbon production on flood tide may be a key element needed for the maintenance of tidal wetland production (Odum et al. 1995). Tidal pulse was determined to be essential for organic carbon production in a series of wetlands in the Hudson River, Florida Everglades and Cooper River Estuary (Romigh et al. 2006; McKellar et al. 2007; Arrigoni et al. 2008). Even though the retention time associated with this daily tidal pulse seems short, it could be essential to freshwater tidal wetlands which function at high frequency time scales due to the influence of daily tide. A few hours difference in the overlap of ebb tide with daylight hours was sufficient to significantly affect chlorophyll *a* flux in the Mildred Island wetland in SFE (Lucas et al. 2006). Small differences in light availability and water temperature were also important for diatom production in Yolo Bypass floodplain in SFE (Lehman et al. 2008).

The vegetated ponds were a relatively large source of material to the freshwater tidal wetland in this study, despite their small size, but information from a much longer time interval is needed to gain a full understanding of the potential magnitude of that contribution due to the high spatial and temporal variability. High spatial variability in water quality conditions characterize freshwater tidal wetlands due to differences in plant associations, elevation and hydrology (Pasternack and Brush 1998; Hein et al. 2004; McKellar et al. 2007) and influence material flux through small scale velocity gradients (Lightbody et al. 2008). In the Cosumnes River and Yolo Bypass floodplains, chlorophyll *a* concentration varied by a factor of 17 over 0.36 km^2^ and water quality conditions varied at the 0.5–1.3 km scale (Ahearn et al. 2006; Sommer et al. 2008). Material flux also varied by a factor of 5 within 1.5 km, a factor of 2–3 daily and by orders of magnitude seasonally in Main Pond between 2004 and 2005 (Lehman et al. 2010). Even greater variation would occur over time due to physical changes in wetland morphometry, tidal hydrography, vegetation type, spring-neap tidal variation and climate change (Pasternack and Brush 1998; Ganju et al. 2005; California Department of Water Resources 2006; Ross et al. 2006; McKellar et al. 2007).

**Figure 10:**
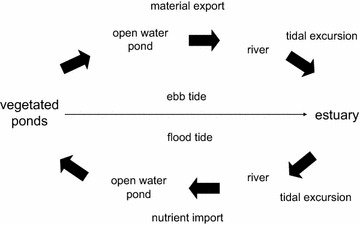
Conceptual model of the influence of tide on daily material flux in Liberty Island wetland.

However, even a small amount of material export from the vegetated ponds may be an important aspect of wetland production, due to the importance of daily tidal excursion. Over 90% of the daily material flux of chlorophyll *a*, total suspended solids and salt in Main Pond was due to tidal dispersion (Lehman et al. 2010). This means that once material from the vegetated ponds is exported into Main Pond on ebb tide, it is subject to a further 6 km tidal excursion out of Main Pond and into the adjacent Sacramento River. The combined tidal flux and excursion from the small vegetated ponds, could then provide food and habitat resources (e.g., phytoplankton, salinity and sediment) to support estuarine production outside of the wetland. It is not necessary for the wetland to have a net export of material on a seasonal or yearly basis for this to occur. The daily movement of material with the tide alone would be sufficient. Tidal excursion may function like a conveyer belt that moves inorganic and organic material from the wetland into the estuary for fishery production on ebb tide and returns nutrients from the estuary to the vegetated ponds for growth of organic matter, reliably two times each day (Figure 10). In fact, the daily tidal flux of materials and nutrients may be more essential to the enhancement of aquatic food webs adjacent to and within wetlands than seasonal or yearly flux, because it works within the life cycle of plankton.

## Conclusion

This study demonstrated the dynamic variation of material flux among vegetated and open water wetland ponds over the tidal day due to material concentration, flow and tide. The study also demonstrated the importance of small vegetated ponds to the organic matter flux of a large wetland. The high variability of inorganic and organic material flux caused by physical factors such as geography, tide and flow lead to the conclusion that understanding material flux in a complex freshwater tidal wetland, such as Liberty Island, requires high frequency spatial and temporal sampling of both large and small pond habitats.

## Methods

### Study area

Liberty Island is a 21 km^2^ freshwater perennial tidal wetland that was created by a levee failure in 1998. A system of levees separates this wetland from the Yolo Bypass floodplain on the north, the Sacramento River on the south, Shag Slough on the west and the Sacramento deep water ship channel on the east (Figure 1). The wetland is called a flooded island because agricultural practices have stripped off the top soil within the levied region so that the surface of the sediment is below sea level. The upper third of the wetland consists of the two shallow ponds, Upper Beaver Pond (1.4 km^2^) and Lower Beaver Pond (5.6 km^2^). These vegetated ponds primarily contain emergent vegetation consisting of tule rush and cattail. Upper Beaver Pond exchanges water with Shag Slough at SSB and Lower Beaver Pond at LIBN (Figure 1). Lower Beaver pond is located in the interior of the wetland and is not directly connected to a river channel, but water exchange occurs with Upper Beaver Pond at LIBN and the open water pond, Main Pond, at LIBS. Main Pond is a large 14 km^2^ open water pond, which comprises two-thirds of the wetland. The southern end of Main Pond at MAIN exchanges water with the Sacramento River. Water is exchanged between all of the ponds, except at low water on ebb tide, when Upper and Lower Beaver Ponds become disconnected due to an elevated portion of land in the middle of Lower Beaver Pond. Water depth in Upper and Lower Beaver Ponds reaches 2 m at high tide. In Main Pond, water depth is less than 1 m at low tide in the north and gradually increases toward the south, where it is 20 m deep. The depth of Main Pond also decreases from 1.5 to 3 m in the west to 0.3 to 2 m in the east.

The flow of water in the wetland is controlled by exchange with the adjacent river channels and a semi-diurnal tide. Average daily discharge in the Sacramento River was 286 ± 3 m^3^ s^−1^ during the 12–13 July 2006 tidal day of this study and was representative of flow during the month, which ranged from 579 to 637 m^3^ s^−1^. Average daily discharge of Shag Slough during the study was 0.0016 ± 0.0003 m^3^ s^−1^ and was orders of magnitude lower than the flow in the Sacramento River.

### Field sampling

Discrete samples were collected at SSB, LIBN, LIBS and MAIN every 1.5 h between 0600 hours on 12 July to 0730 hours on 13 July; 16–18 discrete water quality measurements per station (Figure 1). Water samples were collected using a van Dorn water sampler, immediately cooled to 4°C and processed within 2 h of collection. Processing times strictly followed Standard Methods (APHA et al. 1998). For chlorophyll *a* and phaeophytin pigment concentration measurements, 250–500 ml of raw water was filtered through a GF/F glass fiber filter to which 1 ml of saturated magnesium carbonate solution was added as a preservative, and filters were frozen until spectrophotometric analysis (APHA et al. 1998). The volume of water filtered was decreased to 250 ml, when elevated suspended sediment clogged the filter. A 40 ml water sample was also filtered through a pre-combusted GF/F glass fiber filter for dissolved organic carbon analysis (APHA et al. 1998).

Water samples (250–500 ml) for dissolved inorganic material analysis including soluble reactive phosphorus, nitrate, ammonia, silica, chloride and bromide were filtered through HA nucleopore filters with a 0.45 µm pore size and the filtrate was either frozen or kept at 4°C until analysis (US EPA 1983; USGS 1985). Unfiltered water for measurement of total and volatile suspended solids (1,000 ml), total organic carbon (40 ml) and total phosphorus analysis (250 ml) was kept at 4°C until processing (APHA et al. 1998). Error is based on 10% replication, which produced an average error of 2% for inorganic and 5% for organic variables.

Water for phytoplankton identification and enumeration was placed in a 50 ml amber glass bottle with 1 ml of Lugol’s solution added as a preservative. Phytoplankton cells were identified, sized and enumerated at 700× using the inverted microscope technique (Utermöhl 1958). Phytoplankton were divided into four groups, which comprised the majority of the cells, diatoms, green algae, cyanobacteria and miscellaneous flagellates. Miscellaneous flagellates included cryptophytes and chrysophytes. The biomass of the phytoplankton cells was computed from cell volume based on cell dimensions applied to simple geometric shapes (Menden-Deuer and Lessard 2000).

Continuous water temperature, specific conductance, dissolved oxygen concentration, pH, nephelometric turbidity units (NTU) and chlorophyll *a* fluorescence were measured with YSI 6600 sondes at each station. Chlorophyll *a* fluorescence and NTU were converted to chlorophyll *a* and total suspended solids concentration using linear regression analysis with discrete sample values.

### Hydrodynamic measurements

Due to differences in terrain, flow was measured differently for each station. At SSB, the flow was computed as the sum of the change in water volume of Upper Beaver Pond measured by pressure sensors in Yellow Springs Harbor 6600 water quality sondes (YSI 6600 sondes) and the flow at LIBN. At LIBN, flow was computed using the index velocity method where water velocity, direction and depth were measured with a sideward looking acoustic Doppler continuous profile flow system (ADCP) deployed from a boat. The water flow was calculated as the mean velocity multiplied by the area of the pond. Hourly changes in water volume of Upper Beaver Pond were calculated as the change in water depth divided by the change in time multiplied by the area. The area of Upper Beaver Pond was variable and adjusted based on the effect of tide on water depth. It was estimated that high tide covered 82.5% of the pond area, while low tide covered approximately 40%. At LIBS, the flow was determined from the empirical relationship between the velocity measured by a fixed depth upward looking shallow water ADCP (SonTek Argonaut) and the total flow.

The Main Pond is virtually a lake with many small breeches in the surrounding levee and is connected to the Sacramento River at MAIN, in the southern portion of the pond. There was no practical way to calculate the flows at each levee breech, therefore the total flow at MAIN was calculated from changes in water volume of Main Pond estimated by the total area and change in depth measured using three pressure gauges in YSI 6600 sondes placed across the middle of the pond. It was estimated that 90% of the land in Main Pond was flooded during high tide and 85% was flooded during low tide. For LIBN, LIBS and MAIN positive water flow values indicated water movement in the wetland from north to south and negative water flow values indicated water movement from south to north. For SSB, positive water flow values indicated water movement from west to east, while negative flow values indicated water movement from east to west.

### Material flux

Material flux was measured at four stations located at water exchange points between Shag Slough and Upper Beaver Pond (SSB), Upper Beaver Pond and Lower Beaver Pond (LIBN), Lower Beaver Pond and Main Pond (LIBS) and Main Pond and the Sacramento River (MAIN; Figure 1). Lidar analysis of the wetland geography indicated these stations were the major water exchange points. Material exchange was estimated from continuous flow measurements and discrete water sample values averaged over 15 min intervals. The daily net change (flux) of each material at each station was computed as the product of the mean flow and the material concentration computed for 15 min intervals summed over the 25.5 h tidal day (n = 102). Negative material flux described the net movement of material northward at LIBN, LIBS and MAIN and westward at SSB. Positive material flux described the net movement of material southward at LIBN, LIBS and MAIN and eastward at SSB. The percentage contribution of each pond to the total flux of each inorganic or organic material within the wetland was computed as the absolute value of the material flux at each pond divided by the sum of the absolute value of the material flux of all ponds.

### Statistical analysis

Due to the lack of normality, associations among data values were determined using nonparametric statistical techniques for single (Wilcoxon) and multiple (Kruskal–Wallis) comparisons (SAS Inc. 2013). Individual data values in the text were expressed as the mean and standard deviation.
